# Reduced occludin and claudin-7 expression is associated with urban locations and exposure to second-hand smoke in allergic rhinitis patients

**DOI:** 10.1038/s41598-020-79208-y

**Published:** 2021-01-13

**Authors:** Siti Muhamad Nur Husna, Che Othman Siti Sarah, Hern-Tze Tina Tan, Norasnieda Md. Shukri, Noor Suryani Mohd Ashari, Kah Keng Wong

**Affiliations:** 1 Department of Immunology, School of Medical Sciences, Universiti Sains Malaysia, 16150 Kubang Kerian, Kelantan Malaysia; 2 Hospital Universiti Sains Malaysia, Jalan Raja Perempuan Zainab II, 16150 Kubang Kerian, Kelantan Malaysia; 3 Department of Otorhinolaryngology, Head and Neck Surgery, School of Medical Sciences, Universiti Sains Malaysia, 16150 Kubang Kerian, Kelantan Malaysia

**Keywords:** Adaptive immunity, Mucosal immunology, Risk factors

## Abstract

The breakdown of nasal epithelial barrier occurs in allergic rhinitis (AR) patients. Impairment of cell junction molecules including tight junctions (TJs) and desmosomes plays causative roles in the pathogenesis of AR. In this study, we investigated the transcript expression levels of TJs including occludin (*OCLN*), claudin-3 and -7 (*CLDN3* and *CLDN7*), desmoglein 3 (*DSG3*) and thymic stromal lymphopoietin (*TSLP*) in AR patients (n = 30) and non-allergic controls (n = 30). Nasal epithelial cells of non-allergic controls and AR patients were collected to examine their mRNA expression levels, and to correlate with clinico-demographical and environmental parameters. We demonstrated that the expression of *OCLN* (*p* = 0.009), *CLDN3* (*p* = 0.032) or *CLDN7* (*p* = 0.004) transcript was significantly lower in AR patients compared with non-allergic controls. No significant difference was observed in the expression of *DSG3* (*p* = 0.750) or *TSLP* (*p* = 0.991) transcript in AR patients compared with non-allergic controls. A significant association between urban locations and lower *OCLN* expression (*p* = 0.010), or exposure to second-hand smoke with lower *CLDN7* expression (*p* = 0.042) was found in AR patients. Interestingly, none of the TJs expression was significantly associated with having pets, frequency of changing bedsheet and housekeeping. These results suggest that defective nasal epithelial barrier in AR patients is attributable to reduced expression of *OCLN* and *CLDN7* associated with urban locations and exposure to second-hand smoke, supporting recent findings that air pollution represents one of the causes of AR.

## Introduction

Epithelial barrier serves as the first line defense of the immune system where an intact mucosal barrier is crucial in protecting the host immune system from the exposure of harmful pathogens. Breakdown of nasal epithelial barrier integrity is attributable to reduced expression of tight junction (TJ) molecules which is observed in patients with allergic diseases such as allergic rhinitis (AR). TJ disruption-inducing factors include air pollutants [*e.g.* diesel exhaust particles (DEPs) and fine particulate matter ≤ 2.5 μm (PM2.5)]^1–3^, house dust mites (HDMs) through their proteolytic activity^4^, and T helper 2 (Th2) cytokines (*i.e.* alter composition and permeability of TJs)^5,6^.

Thymic stromal lymphopoietin (TSLP) is a hallmark feature in allergic inflammatory diseases such as asthma and AR by potently deregulating Th2 responses as well as regulating epithelial barrier integrity. AR is a common disease affecting approximately 400 million people worldwide^7,8^. Although AR is not usually a severe disease, it significantly impairs the quality of life (QOL), school or work performance, and with high cost of treatment, leading to major social-economic consequences, and the disease is usually accompanied with comorbidities such as asthma, conjunctivitis and sinusitis^9^. Recently, it has been observed that impairment of nasal epithelial barrier is one of the underlying causes of AR^10,11^. Decreased expression of TJ molecules were observed in AR patients compared with non-allergic controls^11^.

Past studies have focused on moderate/severe HDM-induced AR patients where the main allergens patients sensitized to are HDMs including *Dermatophagoides pteronyssinus* (*D. pteronyssinus*)*, Dermatophagoides farinae* (*D. farinae*) and *Blomia tropicalis* (*B. tropicalis)*. Around 57–80% of AR patients in Malaysia are sensitized to these species^12–15^. HDM allergen is also highly associated with the disruption of epithelial barrier where they have proteolytic activity that can cleave the epithelial TJ proteins. *Der p* 1 (*i.e.* a HDM cysteine proteinase allergen) has been reported to cleave extracellular domain sites in OCLN and in CLDN1, resulted in amplified epithelial permeability that allowed the passage of *Der p* 1 through the epithelial barrier^4,16,17^. There is a lack of literature on the TJs and no study of desmosomal cadherin desmogleins (DSGs) expression in HDM-induced AR. Moreover, data associating TJs expression and environmental factors such as urban versus rural locations and exposure to second-hand smoke remain scarce. This is pivotal as air pollution has been reported to play causative roles in the onset of AR^18–20^.

Targeting the nasal epithelial barrier through restoring the expression and function of TJ and DSG molecules, as well as through regulation by TSLP, may represent a novel approach in developing targeted therapies for AR. Thus, our study was undertaken to investigate the potential association of TSLP with the levels of TJ and DSG molecules in the nasal epithelial cells of HDM-induced AR patients compared with non-allergic controls. Associations between these molecules’ expression and clinico-demographical or environmental factors were also examined.

## Results

### Demographic, clinical characteristics and environmental factors of non-allergic controls and AR patients

The demographic, clinical characteristics and environmental factors of non-allergic controls and AR patients are presented in Table 1. Non-allergic controls and AR patients were mostly female but there was no significant difference between sex, age or BMI in non-allergic individuals and AR patients. Majority of AR patients (83.3%) had family history of allergic diseases while none in controls. The highest comorbidity associated with AR was rhinosinusitis (93.3%) followed by pharyngitis (53.3%), conjunctivitis (40.0%), asthma (36.7%) and others (Table 1). AR patients assessed in this study had moderate/severe AR with 70.0% and 30.0% having persistent and intermittent symptoms, respectively.

**Table 1:** Demographic, clinical characteristics and environmental factors of non-allergic controls and AR patients.

	Non-allergic controls (n = 30)	AR patients (n = 30)	*p-*value
**Demographic and clinical characteristics**^**a**^			
Mean age (years) ± SD	31.3 ± 8.164	28.7 ± 8.660	0.066^b^
Sex			
Male	8 (26.7)	10 (33.3)	0.573^c^
Female	22 (73.3)	20 (66.7)	
Mean BMI (kg/m^2^) ± SD	24.2 ± 4.385	26.1 ± 4.942	0.065^b^
Family history of allergic diseases			
Yes	0 (0.0)	25 (83.3)	–
No	30 (100.0)	5 (16.7)	
Comorbidity			
Rhinosinusitis	NA	28 (93.3)	–
Pharyngitis	NA	16 (53.3)	–
Conjunctivitis	NA	12 (40.0)	–
Asthma	NA	11 (36.7)	–
Otitis media	NA	5 (16.7)	–
Obstructive sleep apnea	NA	1 (3.3)	–
Symptoms classification			
Intermittent	NA	9 (30.0)	–
Persistent	NA	21 (70.0)	–
**Environmental factors**			
Exposure to secondhand smoke			
Yes	12 (40.0)	21 (70.0)	**0.020**^**c**^
No	18 (60.0)	9 (30.0)	
Home location			
Urban	13 (43.3)	17 (56.7)	0.302^c^
Rural	17 (56.7)	13 (43.3)	
Having pets			
Yes	18 (60.0)	16 (53.3)	0.603^c^
No	12 (40.0)	14 (46.7)	
Frequency of changing bed sheets and pillowcase			
Weekly	12 (40.0)	12 (40.0)	1.000^d^
Monthly	15 (50.0)	14 (46.7)	
2-Monthly	3 (10.0)	4 (13.3)	
Frequency of doing housekeeping			
Daily	15 (50.0)	10 (33.3)	**0.016**^**d**^
Weekly	15 (50.0)	13 (43.3)	
Alternate day	0 (0.0)	7 (23.3)	

Abbreviations: AR, allergic rhinitis; BMI, body mass index; SD, standard deviation; NA, not applicable.

^a^Data are presented as number (percentage) unless stated otherwise.

^b^Mann–Whitney test.

^c^Chi-square test.

^d^Fisher’s exact test.

In terms of comparison of environmental factors between non-allergic controls and AR patients, there was a significant difference in the exposure to second-hand smoke between non-allergic controls and AR patients (*p* = 0.020) with nearly 2 times more AR patients exposed to second-hand smoke. A significant difference was also observed in the frequency of performing housekeeping tasks where AR patients performed housekeeping less frequently than controls (*p* = 0.016). Other factors such as home location (*i.e.* urban and rural), pets, frequency of changing bed sheets and pillowcase did not show any significant difference between the two study populations.

### Sensitization to HDM allergens

In this study, AR patients were tested for sensitization to the HDM allergens *B. tropicalis*, *D. farinae* and *D. pteronysinnus*. All AR patients showed sensitization to at least one of the HDM allergens tested. A total of 83.3% (n = 25) sensitized to *D. farinae*, and 66.7% (n = 20) of the patients sensitized to both *B. tropicalis* and *D. pteronysinnus*. Finally, 46.7% (n = 14) of the patients sensitized to all HDM allergens tested in this study.

### Assessment of symptoms severity and quality of life (QOL) scores in AR patients

Supplementary Table S1 shows the median severity scores of nasal and non-nasal symptoms in this cohort of AR patients. In assessing nasal symptoms severity scores, all AR patients reported that they were moderately troubled by sneezing, runny nose, congestion (stuffiness) and itchy nose symptoms. Furthermore, they were mildly affected by postnasal drip symptoms. In addition, for non-nasal symptoms, all moderate/severe AR patients had mild eye or ear symptoms, headache and also mental function (cognitive impairment), as well as mild throat symptoms and occasional chronic cough. The effects of nasal and non-nasal symptoms or rhinitis severity on QOL scores (median) in AR patients are presented in Supplementary Table S2. In the QOL assessment of nasal and non-nasal symptoms or rhinitis severity, the patients were frequently affected by sleep disturbance at night, impairment of work performance, impairment of social and/or recreational activities.

### Expression of *OCLN*, *CLDN3*, *CLDN7*,* DSG3* or* TSLP* transcript in non-allergic controls and AR patients

**Figure 1:**
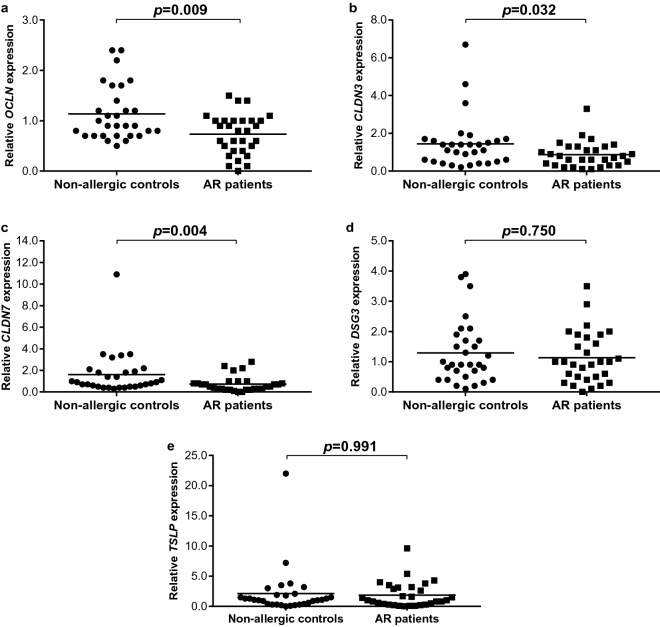
Relative *OCLN* (**a**), *CLDN3* (**b**)*, CLDN7* (**c**)*, DSG3* (**d**) and *TSLP* (**e**) expression in non-allergic controls (n = 30) and AR patients (n = 30). Bar represents mean.

The expression of *OCLN* (*p* = 0.009), *CLDN3* (*p* = 0.032) or *CLDN7* (*p* = 0.004) transcript was significantly lower in AR patients compared with non-allergic controls. No significant difference was observed in the expression of *DSG3* (*p* = 0.750) or *TSLP* (*p* = 0.991) transcript in AR patients compared with non-allergic controls (Fig. 1).

### Association of *OCLN*, *CLDN3*, *CLDN7*, *DSG3* and *TSLP* expression with clinico-demographical parameters of AR patients and non-allergic controls

The associations of *OCLN*, *CLDN3*, *CLDN7*, *DSG3* and *TSLP* expression in AR patients with demographical and clinical parameters of AR patients are shown in Tables 2 and 3, respectively. These associations were also investigated in non-allergic controls as shown in Table 4.

**Table 2:** Association of *OCLN*, *CLDN3, CLDN, DSG3* and *TSLP* expression with demographical parameters of AR patients (n = 30).

	n (%)	*OCLN* expression			*CLDN3* expression			*CLDN7* expression			*DSG3* expression			*TSLP* expression		
		< Median	≥ Median	*p*-value	< Median	≥ Median	*p*-value	< Median	≥ Median	*p*-value	< Median	≥ Median	*p*-value	< Median	≥ Median	*p*-value
**Age (years)**																
Median (range)	30 (20–54)															
< 27.5	15 (50.0)	7 (23.3)	8 (26.7)	1.000	8 (26.7)	7 (23.3)	0.715	7 (23.3)	8 (26.7)	0.712	10 (33.3)	5 (16.7)	**0.028**	8 (26.7)	7 (23.3)	0.464
≥ 27.5	15 (50.0)	7 (23.3)	8 (26.7)		7 (23.3)	8 (26.7)		6 (20.0)	9 (30.0)		4 (13.3)	11 (36.7)		6 (20.0)	9 (30.0)	
**BMI**																
Median (range)	30 (19.0–34.02)															
< 26.5	15 (50.0)	11 (36.7)	4 (13.3)	**0.003**	8 (26.7)	7 (23.3)	0.715	7 (23.3)	8 (26.7)	0.712	8 (26.7)	7 (23.3)	0.464	7 (23.3)	8 (26.7)	1.000
≥ 26.5	15 (50.0)	3 (10.0)	12 (40.0)		7 (23.3)	8 (26.7)		6 (20.0)	9 (30.0)		6 (20.0)	9 (30.0)		7 (23.3)	8 (26.7)	
**Sex**																
Male	10 (33.3)	4 (13.3)	6 (20.0)	0.709 (F)	4 (13.3)	6 (20.0)	0.434	5 (16.7)	5 (16.7)	0.705 (F)	5 (16.7)	5 (16.7)	1.000 (F)	6 (20.0)	4 (13.3)	0.442 (F)
Female	20 (66.7)	10(33.3)	10 (33.3)		11 (36.7)	9 (30.0)		8 (26.7)	12 (40.0)		9 (30.0)	11 (36.7)		8 (26.7)	12 (40.0)	
**Exposure to secondhand smoke**																
Yes	21 (70.0)	8 (26.7)	13 (43.3)	0.236 (F)	9 (30.0)	12 (40.0)	0.427 (F)	12 (40.0)	9 (30.0)	**0.042 (F)**	10 (33.3)	11 (36.7)	1.000 (F)	12 (40.0)	9 (30.0)	0.118 (F)
No	9 (30.0)	6 (20.0)	3 (10.0)		6 (20.0)	3 (10.0)		1 (3.3)	8 (26.7)		4 (13.3)	5 (16.7)		2 (6.7)	7 (23.3)	
**Home location**																
Urban	16 (53.3)	11 (36.7)	5 (16.7)	**0.010**	9 (30.0)	7 (23.3)	0.464	6 (20.0)	10 (33.3)	0.491	7 (23.3)	9 (30.0)	0.732	10 (33.3)	6 (20.0)	0.063
Rural	14 (46.7)	3 (10.0)	11 (36.7)		6 (20.0)	8 (26.7)		7 (23.3)	7 (23.3)		7 (23.3)	7 (23.3)		4 (13.3)	10 (33.3)	
**Having pets**																
Yes	17 (56.7)	8 (26.7)	9 (30.0)	0.964	8 (26.7)	9 (30.0)	0.712	5 (16.6)	12 (40.0)	0.078	10 (33.3)	7 (23.3)	0.127	10 (33.3)	7 (23.3)	0.127
No	13 (43.3)	6 (20.0)	7 (23.3)		7 (23.3)	6 (20.0)		8 (26.7)	5 (16.7)		4 (13.3)	9 (30.0)		4 (13.3)	9 (30.0)	
**Frequency of changing bedsheet**																
Weekly	12 (40.0)	6 (20.0)	6 (20.0)	0.363 (F)	5 (16.7)	7 (23.3)	0.877 (F)	4 (13.3)	8 (26.7)	0.414 (F)	5 (16.7)	7 (23.3)	0.686 (F)	5 (16.7)	7 (23.3)	0.686 (F)
Monthly	14 (46.7)	5 (16.7)	9 (30.0)		8 (26.7)	6 (20.0)		8 (26.7)	6 (20.0)		8 (26.7)	6 (20.0)		8 (26.7)	6 (20.0)	
2-Monthly	4 (13.3)	3 (10.0)	1 (3.3)		2 (6.7)	2 (6.7)		1 (3.3)	3 (10.0)		1 (3.3)	3 (10.0)		1 (3.3)	3 (10.0)	
**Frequency of housekeeping**																
Daily	11 (36.7)	5 (16.7)	6 (20.0)	0.095 (F)	4 (13.3)	7 (23.3)	0.642 (F)	5 (16.7)	6 (20.0)	1.000 (F)	5 (16.7)	6 (20.0)	0.468 (F)	5 (16.7)	6 (20.0)	0.468 (F)
Weekly	12(40.0)	8 (26.7)	4 (13.3)		7 (23.3)	5 (16.7)		5 (16.7)	7 (23.3)		7 (23.3)	5 (16.7)		7 (23.3)	5 (16.7)	
Alternate day	7 (23.3)	1(3.3)	6 (20.0)		4 (13.3)	3 (10.0)		3 (10.0)	4 (13.3)		2 (6.7)	5 (16.7)		2 (6.7)	5 (16.7)	

*p* < 0.05 shown in bold. Abbreviation: AR, allergic rhinitis; *OCLN*, occludin; *CLDN3*, claudin-3; *CLDN7*, claudin-7; *DSG3*, desmoglein 3; *TSLP*, thymic stromal lymphopoietin; BMI, body mass index.

**Table 3:** Association of *OCLN*, *CLDN3, CLDN, DSG3* and *TSLP* expression with clinical parameters of AR patients (n = 30).

	n (%)	*OCLN* expression			*CLDN3* expression			*CLDN7* expression			*DSG3* expression			*TSLP* expression		
		< Median	≥ Median	*p*-value	< Median	≥ Median	*p*-value	< Median	≥ Median	*p*-value	< Median	≥ Median	*p*-value	< Median	≥ Median	*p*-value
**Family History of Allergic Diseases**																
Yes	25 (83.3)	13 (43.3)	12 (40.0)	0.336 (F)	13 (43.3)	12 (40.0)	1.000 (F)	12 (40.0)	13 (43.3)	0.355 (F)	11 (36.7)	14 (46.7)	0.642 (F)	10 (33.3)	15 (50.0)	0.157 (F)
No	5 (16.7)	1 (3.3)	4 (13.3)		2 (6.7)	3 (10.0)		1(3.3)	4 (13.3)		3 (10.0)	2 (6.7)		4 (13.3)	1 (3.3)	
**Classification of AR**																
Persistent	21 (70.0)	9 (30.0)	12 (40.0)	0.694 (F)	12 (40.0)	9 (30.0)	0.427 (F)	8 (26.7)	13 (43.3)	0.443 (F)	10 (33.3)	11 (36.7)	1.000 (F)	11 (36.7)	10 (33.3)	1.000 (F)
Intermittent	9 (30.0)	5 (16.7)	4 (13.3)		3 (10.0)	6 (20.0)		5 (16.7)	4 (13.3)		4 (13.3)	5 (16.7)		5 (16.7)	4 (13.3)	
**Conjunctivitis**																
Yes	22 (73.3)	9 (30.0)	13 (43.3)	0.417 (F)	12 (40.0)	10 (33.3)	0.682 (F)	8 (26.7)	14 (46.7)	0.242 (F)	10 (33.3)	12 (40.0)	1.000 (F)	10 (33.3)	12 (40.0)	1.000 (F)
No	8 (26.7)	5 (16.7)	3 (10.0)		3 (10.0)	5 (16.7)		5 (16.7)	3 (10.0)		4 (13.3)	4 (13.3)		4 (13.3)	4 (13.3)	
**Pharyngitis**																
Yes	16 (53.3)	4 (13.3)	12 (40.0)	**0.011**	8 (26.7)	7 (23.3)	0.715	7 (23.3)	8 (26.7)	0.712	8 (26.7)	8 (26.7)	0.696	7 (23.3)	9 (30.0)	0.732
No	14 (46.7)	10 (33.3)	4 (13.3)		7 (23.3)	8 (26.7)		6 (20.0)	9 (30.0)		6 (20.0)	8 (26.7)		7 (23.3)	7 (23.3)	
**Asthma**																
Yes	11 (36.7)	4 (13.3)	7 (23.3)	0.389	6 (20.0)	5 (16.7)	0.704	4 (13.3)	7 (23.3)	0.708 (F)	5 (16.7)	6 (20.0)	0.920	3 (10.0)	8 (26.7)	0.105
No	19 (63.3)	10 (33.3)	9 (30.0)		9 (30.0)	10 (33.3)		9 (30.0)	10 (33.3)		9 (30.0)	10 (33.3)		11 (36.7)	8 (26.7)	
**Sinusitis**																
Yes	28 (93.3)	12 (40.0)	16 (53.3)	0.209 (F)	14 (46.7)	14 (46.7)	1.000 (F)	11 (36.7)	17 (56.7)	0.179 (F)	14 (46.7)	14 (46.7)	0.485 (F)	13 (43.3)	15 (50.0)	1.000 (F)
No	2 (6.7)	2 (6.7)	0 (0.0)		1 (3.3)	1 (3.3)		2 (6.7)	0 (0.0)		0 (0.0)	2 (6.7)		1 (3.3)	1 (3.3)	
**Otitis Media**																
Yes	5 (16.7)	2 (6.7)	3 (10.0)	1.000 (F)	1 (3.3)	4 (13.3)	0.330 (F)	4 (13.3)	1 (3.3)	0.128 (F)	2 (6.7)	3 (10.0)	1.000 (F)	1 (3.3)	4 (13.3)	0.336 (F)
No	25 (83.3)	12 (40.0)	13 (43.3)		14 (46.7)	11 (36.7)		8 (26.7)	17 (56.7)		12 (40.0)	13 (43.3)		13 (43.3)	12 (40.0)	
**Sensitivity to HDM allergen**																
***D. farina***																
Yes	24 (80.0)	11 (36.7)	13 (43.3)	1.000 (F)	13 (43.3)	11 (36.7)	0.651 (F)	11 (36.7)	13 (43.3)	0.672 (F)	11 (36.7)	13 (43.3)	1.000 (F)	11 (36.7)	13 (43.3)	1.000 (F)
No	6 (20.0)	3 (10.0)	3 (10.0)		2 (6.7)	4 (13.3)		2 (6.7)	4 (13.3)		3 (10.0)	3 (10.0)		3 (10.0)	3 (10.0)	
***D. pteronysinnus***																
Yes	19 (63.3)	9 (30.0)	10 (33.3)	0.920	9 (30.0)	10 (33.3)	0.704	11 (36.7)	8 (26.7)	0.057 (F)	6 (20.0)	13 (43.3)	**0.030**	9 (30.0)	10 (33.3)	0.920
No	11 (36.7)	5 (16.7)	6 (20.0)		6 (20.0)	5 (16.7)		2 (6.7)	9 (30.0)		8 (26.7)	3 (10.0)		5 (16.7)	6 (20.0)	
***B. tropicalis***																
Yes	21 (70.0)	10 (33.3)	11 (36.7)	1.000 (F)	12 (40.0)	9 (30.0)	0.427 (F)	7 (23.3)	14 (46.7)	0.123 (F)	11 (36.7)	10 (33.3)	0.440 (F)	9 (30.0)	12 (40.0)	0.694
No	9 (30.0)	4 (13.3)	5 (16.7)		3 (10.0)	6 (20.0)		6 (20.0)	3 (10.0)		3 (10.0)	6 (20.0)		5 (16.7)	4 (13.3)	

*p* < 0.05 shown in bold. Abbreviation: AR, allergic rhinitis; *OCLN*, occludin; *CLDN3*, claudin-3; *CLDN7*, claudin-7; *DSG3*, desmoglein 3; *TSLP*, thymic stromal lymphopoietin; HDM, house dust mite.

**Table 4:** Association of *OCLN, CLDN3* and *CLDN7, DSG3* and *TSLP* expression with demographical parameters of non-allergic individuals (n = 30).

	n (%)	*OCLN* expression			*CLDN3* expression			*CLDN7* expression			*DSG3* expression			*TSLP* expression		
		< Median	≥ Median	*p*-value	< Median	≥ Median	*p*-value	< Median	≥ Median	*p*-value	< Median	≥ Median	*p*-value	< Median	≥ Median	*p*-value
**Age (years)**																
Median (range)	30 (24–54)															
< 28	12 (40.0)	4 (13.3)	8 (26.7)	0.709 (F)	6 (20.0)	6 (20.0)	1.000	4 (13.3)	8 (26.7)	0.136	8 (26.7)	4 (13.3)	**0.024 (F)**	6 (20.0)	6 (20.0)	0.765
≥ 28	18 (60.0)	8 (26.7)	10 (33.3)		9 (30.0)	9 (30.0)		11 (36.7)	7 (23.3)		4 (13.3)	14 (46.7)		8 (26.7)	10 (33.3)	
**BMI**																
Median (range)	30 (18.59–37.39)															
< 22.86	15 (50.0)	10 (33.3)	5 (16.7)	**0.003**	9 (30.0)	6 (20.0)	0.273	9 (30.0)	6 (20.0)	0.273	8 (26.7)	7 (23.3)	0.136	9 (30.0)	6 (20.0)	0.143
≥ 22.86	15 (50.0)	2 (6.7)	13 (43.3)		6 (20.0)	9 (30.0)		6 (20.0)	9 (30.0)		4 (13.3)	11 (36.7)		5 (16.7)	10 (33.3)	
**Sex**																
Male	8 (26.7)	3 (10.0)	5 (16.7)	1.000 (F)	3 (10.0)	5 (16.7)	0.682 (F)	4 (13.3)	4 (13.3)	1.000 (F)	3 (10.0)	5 (16.7)	1.000 (F)	3 (10.0)	5 (16.7)	0.689 (F)
Female	22 (733)	9 (30.0)	13 (43.3)		12 (40.0)	10 (33.3)		11 (36.7)	11 (36.7)		9 (30.0)	13 (43.3)		11 (36.7)	11 (36.7)	
**Exposure to secondhand smoke**																
Yes	12 (40.0)	3 (10.0)	9 (30.0)	0.260 (F)	7 (23.3)	5 (16.7)	0.456	6 (20.0)	6 (20.0)	1.000	6 (20.0)	6 (20.0)	0.458 (F)	7 (23.3)	5 (16.7)	0.296
No	18 (60.0)	9 (30.0)	9 (30.0)		8 (26.7)	10 (33.3)		9 (30.0)	9 (30.0)		6 (20.0)	12 (40.0)		7 (23.3)	11 (36.7)	
**Home location**																
Urban	13 (43.3)	3 (10.0)	10 (33.3)	0.098	4 (13.3)	9 (30.0)	0.065	5 (16.7)	8 (26.7)	0.269	6 (20.0)	7 (23.3)	0.547	4 (13.3)	9 (30.0)	0.127
Rural	17 (56.7)	9 (30.0)	8 (26.7)		11 (36.7)	6 (20.0)		10 (33.3)	7 (23.3)		6 (20.0)	11 (36.7)		10 (33.3)	7 (23.3)	
**Having pets**																
Yes	18 (60.0)	7 (23.3)	11 (36.7)	0.879	9 (30.0)	9 (30.0)	1.000	11 (36.7)	7 (23.3)	0.052	6 (20.0)	12 (40.0)	0.361	10 (33.3)	8 (26.7)	0.232
No	12 (40.0)	5 (16.7)	7 (23.3)		6 (20.0)	6 (20.0)		3 (10.0)	9 (30.0)		6 (20.0)	6 (20.0)		4 (13.3)	8 (26.7)	
**Frequency of changing bedsheet**																
Weekly	12 (40.0)	3 (10.0)	9 (30.0)	0.375 (F)	4 (13.3)	8 (26.7)	0.420 (F)	6 (20.0)	6 (20.0)	0.258 (F)	3 (10.0)	9 (30.0)	0.099 (F)	5 (16.7)	7 (23.3)	0.877 (F)
Monthly	15 (15.0)	7 (23.3)	8 (26.7)		9 (30.0)	6 (20.0)		9 (30.0)	6 (20.0)		6 (20.0)	9 (30.0)		8 (26.7)	7 (23.3)	
2-Monthly	3 (10.0)	2 (6.7)	1 (3.3)		2 (6.7)	1 (3.3)		0 (0.0)	3 (10.0)		3 (10.0)	0 (0.0)		1 (3.3)	2 (6.7)	
**Frequency of housekeeping**																
Daily	15 (50.0)	6 (20.0)	9 (30.0)	1.000	9 (30.0)	6 (20.0)	0.273	5 (16.7)	10 (33.3)	0.456	8 (26.7)	7 (23.3)	0.464	5 (16.7)	10 (33.3)	0.456
Weekly	15 (50.0)	6 (20.0)	9 (30.0)		6 (20.0)	9 (30.0)		7 (23.3)	8 (26.7)		6 (20.0)	9 (30.0)		7 (23.3)	8 (26.7)	

*p* < 0.05 shown in bold. Abbreviation: AR, allergic rhinitis; *OCLN*, occludin; *CLDN3*, claudin-3; *CLDN7*, claudin-7; *DSG3*, desmoglein 3; *TSLP*, thymic stromal lymphopoietin; BMI, body mass index.

AR patients with lower BMI (median cut-off; < 26.53) had significantly lower *OCLN* expression (*p* = 0.003). AR patients who lived in urban locations had significantly lower *OCLN* expression (*p* = 0.010) contrasted to those who lived in rural areas (Table 2). Moreover, *OCLN* expression was significantly higher in AR patients with pharyngitis (*p* = 0.011) compared with patients without pharyngitis (Table 3). AR patients exposed to second-hand smoke had significantly lower *CLDN7* expression (*p* = 0.042) compared with AR patients not exposed to second-hand smoke (Table 2), and such association was not observed in non-allergic individuals (Table 4).

Younger AR patients (median cut-off; < 27.5 years old) had a significantly lower *DSG3* expression (*p* = 0.028) (Table 2). Interestingly, AR patients sensitized to *D. pteronysinnus* had significantly higher *DSG3* expression (*p* = 0.030) (Table 3)*.* None of the clinico-demographical parameters showed a significant association with *TSLP* and *CLDN3* expression in AR patients (Tables 2 and 3) or non-allergic controls (Table 4). Non-allergic controls with higher median BMI (median cut-off; ≥ 22.86) showed significantly higher *OCLN* expression (*p* = 0.003), while younger non-allergic controls (median cut-off; < 27.5 years old) also demonstrated a significant association with higher *DSG3* expression (*p* = 0.024).

## Discussion

Accumulating evidence has established that the prevalence of adult AR patients is approximately equal between males and females^21^. For instance, in a meta-analysis of 7 studies consisting of adult AR patients (20,398 males and 23,690 females) aged 18 years and older, no sex-specific prevalence difference was observed whereby the male–female ratio was 0.96 (95% confidence interval 0.83–1.17)^22^. Likewise, there was no statistically significant difference in the distribution of gender between non-allergic controls and AR patients in our study (*p* = 0.573; Table 1) all of whom were adults aged 18 years and older. Taken together, gender distribution in our cohort of adult subjects unlikely played a significant role in our subsequent observations.

In this study, *OCLN* expression was lower in AR patients compared with non-allergic controls consistent with independent findings. Lower *OCLN* mRNA expression and relatively weak arrangement of OCLN protein in biopsy specimens were observed in HDM-induced AR patients compared with control subjects^4^. A loose arrangement of OCLN is attributable to reduced *OCLN* expression and this could expedite the passage of allergens through nasal epithelial barrier as OCLN is localized at the uppermost layer of pseudostratified columnar epithelium of the nasal mucosa. Lower *OCLN* mRNA expression occurred in the nasal biopsies of AR patients compared with healthy subjects and idiopathic rhinitis patients, and a severely disrupted layer and irregular pattern of OCLN expression was observed in the biopsy specimens of AR patients^11^. Furthermore, HLA-DR- and CD11c-positive DCs penetrated beyond OCLN in the epithelium of the nasal mucosa of AR patients^23^. In vivo model of HDM-induced allergic airway inflammation in mice also showed decreased *OCLN* expression^11^.

We observed that *CLDN3* expression was decreased in AR patients compared with non-allergic controls. Moreover, *CLDN7* expression in AR patients was also reduced compared with non-allergic controls in this study. This is comparable with previous studies where *CLDN7* mRNA expression was decreased in nasal mucosa of AR patients^23^. The TJ molecules OCLN, CLDN3 and CLDN7 are located at the apical junction of the epithelial and these TJ molecules are thus more prone to damage caused by allergens. They are also essential in creating rate-limiting barrier to inhaled pathogens. Both CLDN3 and CLDN7 are sealing (or barrier forming) claudins, and they act by reducing the permeability of the epithelial^24,25^.

On the other hand, the expression of *DSG3* showed no significant difference in both study populations, and relatively high levels of *DSG3* expression were observed in AR patients and non-allergic controls. This might be due to localization of DSG3 at the basal epithelial that renders it a lower likelihood to be exposed to allergens, and DSG proteins may not be primary targets for expression downregulation in AR. Recently, dual roles of DSG3 in AR pathogenesis and severity have been shown in AR mice model. Silencing of *DSG3* (siRNA-*DSG3*) showed an alleviation of nasal mucosa inflammation in AR mice model with significantly increased scores of nasal itching, sneezing and rhinorrhea compared with control group (*i.e.* AR mice model without *DSG3* gene silencing)^26^. The nasal mucosa structure of the siRNA-*DSG3* group was more loose and disordered, and the goblet cells were more proliferated compared with the control group. These suggest that *DSG3* gene silencing promotes AR pathogenesis through breakdown of nasal mucosa structure but DSG3 expression contributed to inflammation of AR in mice model.

In the present study, *TSLP* expression in both study populations showed no significant difference as its expression in both AR patients and non-allergic controls was high. Our findings were not comparable with previous studies in terms of *TSLP* expression in AR versus non-allergic controls. Kamekura *et al*. reported that *TSLP* mRNA expression was significantly increased in the nasal mucosa of AR patients compared with controls^27^. *TSLP* was also significantly upregulated in sensitized and nasally-challenged mouse model of AR^28,29^. Moreover, TSLP was responsible for the induction of sneezing responses and increased serum ragweed-specific IgE levels in acute and chronic AR mice model compared with wild type mice^30^.

TSLP is an epithelium cell-derived cytokine^31^ and increased TSLP expression can be attributable to stimulation of nasal epithelial cells to release TSLP after exposure to allergens^29^. In this study, SPT was performed on AR patients and non-allergic controls before samples were collected in order to determine their allergic status. During SPT, when relevant allergens are presented into the skin, specific IgE bound to the surface receptors on mast cells are cross-linked and this causes the degranulation of mast cells and release of other mediators^32^. Although SPT may contribute to altered *TSLP* expression in the nasal epithelial cells of both control and AR patient populations, the time interval between SPT procedure and nasal epithelial cell sampling was under an hour in our study. Changes to sinonasal epithelium integrity require direct exposure of the epithelium to HDM allergens for approximately 24 hours^33^. Moreover, SPT has been known to be safe and systemic side effects are rare^32^. None of our study subjects experienced unusual or nasal-related side effects post-SPT procedures, and the SPT was performed on the forearm of our participants, relatively distant from the site of nasal epithelial cells collection.

There was a significant association between urban locations and lower *OCLN* expression in AR patients. Greater air pollution is known to occur in urban locations compared with rural areas. Sinonasal diseases can be caused by the defect of epithelial barrier due to air pollutants such as DEPs and fine PM2.5^1–3^. The exposure of DEPs to pulmonary neuroendocrine cells in air–liquid interface culture and exposure of PM2.5 to human nasal epithelial cell line (RPMI 2650 cells) significantly reduced the expression of TJ molecules including *OCLN,* zonula occludens-1 (*ZO-1*) and *CLDN1*^1–3^. Permeability of nasal epithelial cells was increased through exposure of DEP mediated by ROS pathway^3^. Loss of barrier function in human nasal epithelium through exposure of PM2.5 also increased the release of proinflammatory cytokines (*i.e.* interleukin-8, TIMP metallopeptidase inhibitor 1 and TSLP)^34^. These suggest an important mechanism of susceptibility to rhinitis in highly PM2.5-polluted areas mainly in urban areas as reported in past studies^35,36^.

Exposure to second-hand smoke in AR patients showed a significant association with lower *CLDN7* expression compared with AR patients not exposed to second-hand smoke. Cigarette smoke is associated with exacerbation of allergic diseases whereby it could disrupt TJ barrier function in human bronchial epithelial cell line (16HBE14o^−^) as well as primary human bronchial epithelial cells^37^. Incubation with cigarette smoke extract reduced TJ proteins (*i.e.* ZO-1 and ZO-2) expression and also caused the dislocation of TJ proteins from the cell membrane^37^. In addition, the stimulation of primary human sinonasal epithelial cells from healthy subjects to cigarette smoke extract decreased ZO-1 and junctional adhesion molecules A (JAMA) expression, and decreased transepithelial resistance levels^38^. The effect of cigarette smoke extract was inhibited by the pharmacologic activation of nuclear factor erythroid 2-related factor 2 (Nrf2) (*i.e.* an anti-oxidant) by enhancing the localization of ZO-1 and JAMA levels at the cell surface, and increased transepithelial resistance levels. This indicates that cigarette smoke extract disrupts TJ through oxidative stress similar with DEP.

We acknowledge the limitations to the current study as follows: (1) We focused on HDM-sensitized AR only without involving other allergen-sensitized AR patients and we thus could not rule out the potential contribution by other allergens to the disruption of cell junction molecules in AR patients; (2) Patients with moderate/severe AR only were recruited in this study, hence we were unable to assess whether alteration of TJs expression may occur in AR patients regardless of disease severity. However, AR patients with HDM allergy typically demonstrate symptoms of moderate/severe rhinitis^39^, and mild AR patients are unlikely to consult a physician due to tolerable symptoms^39,40^.

In conclusion, we have demonstrated for the first time the expression profile of *CLDN3* and *DSG3* in AR compared with non-allergic subjects, and the association of the clinico-demographic parameters of AR and non-allergic subjects with the expression of TJ molecules. Impairment in the nasal epithelial barriers of AR patients is associated with lower *OCLN*, *CLDN3* and *CLDN7* expression. No significant association was observed in *DSG3* expression between two study populations*,* and this molecule might not represent an essential target for expression downregulation in AR. Collectively, reduced expression of OCLN and CLDN molecules in AR might be attributable to living in urban locations and exposure to second-hand smoke. Our data support recent findings that air pollution represents one of the causes of AR, potentially through decreased expression of TJs leading to breakdown of nasal epithelial barrier in the disease.

## Methods

### Patients recruitment

**Figure 2:**
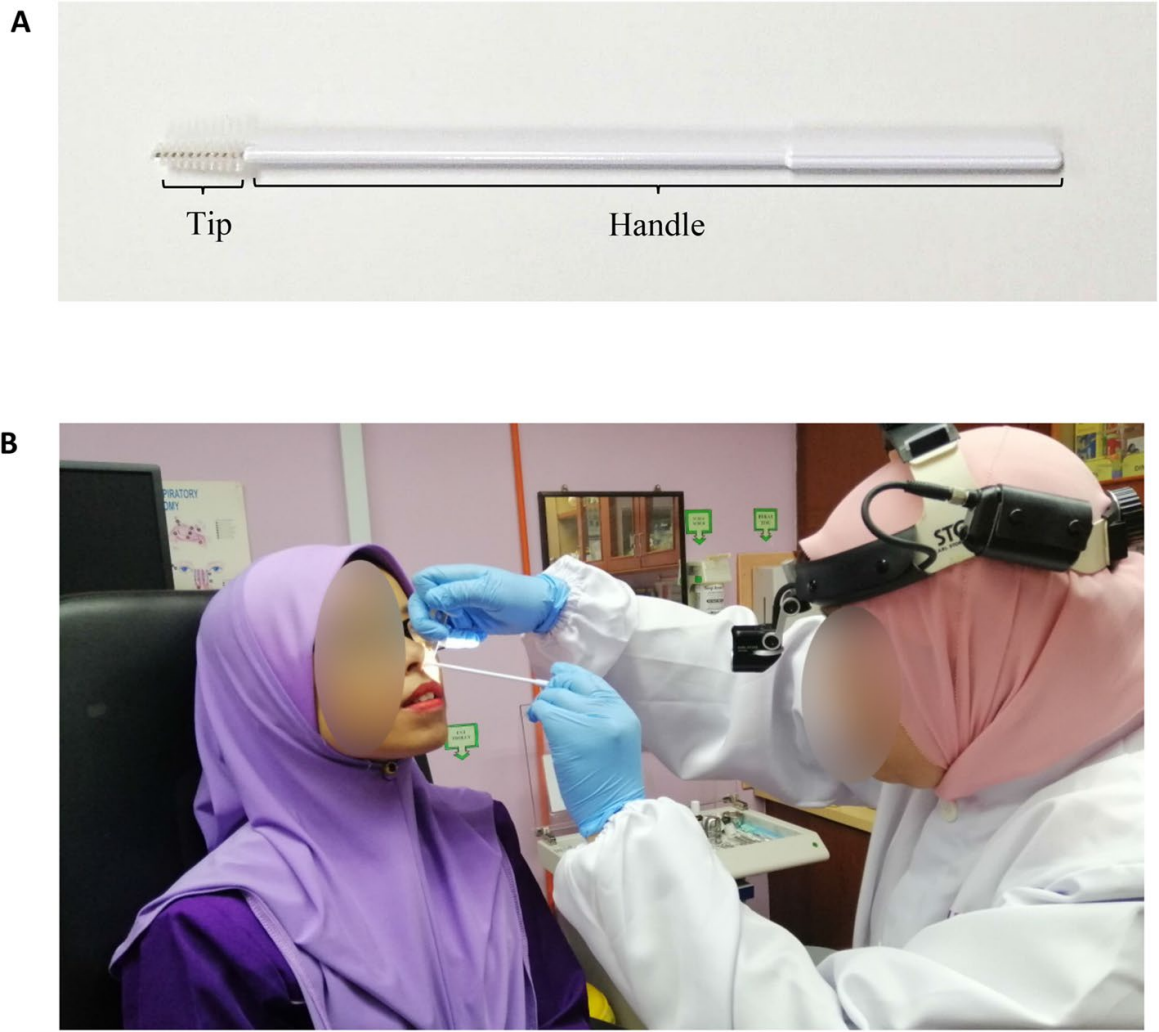
(**A**) Cytology brush used for nasal epithelial cells sample collection. Its tip is 6.5 mm in width and 18 mm in length. The handle is 27 mm in length and diameter of 2.8 mm. (**B**) Picture of study’s subject during nasal brushing procedures by an authorized physician, and the complete procedures were demonstrated in a video (Supplementary Video S1).

All samples were obtained with signed consent under an approved protocol from the Human Research Ethics Committee of Universiti Sains Malaysia (JEPeM) (approved ethics code: USM/JEPeM/18060273). All AR patients and non-allergic individuals participated in this study provided written informed consent for this study. The individuals shown in Fig. 2B and Supplementary Video S1 had given informed consent for both study participation and publication of identifying images in an online open-access publication. All samples were labeled anonymously, and all data were recorded, kept and analyzed anonymously to ensure none of the private information (*e.g.* patient name, gender or age) was disclosed. All procedures conducted involving human participants were according to institutional ethical standards and with the 1964 Declaration of Helsinki and its later updates or comparable ethical standards. All experimental procedures were carried out in accordance with the institutional guidelines and regulations.

This study was conducted in Otorhinolaryngology, Head and Neck Surgery (ORL-HNS) clinic in Hospital Universiti Sains Malaysia (HUSM), and Department of Immunology, School of Medical Sciences, Universiti Sains Malaysia. This study was a cross-sectional study between AR patients as cases and non-allergic participants as controls. The cases of this study were AR patients attending ORL-HNS clinic in HUSM. The controls of this study were non-allergic participants recruited among students, staff members of HUSM and members of the local community who were eligible and willing to participate. The cases and controls of this study were adults (≥ 18 years old) AR patients and non-allergic participants who fulfilled the inclusion criteria and had none of the exclusion criteria of this study (Table 5).

**Table 5:** Inclusion and exclusion criteria of cases and controls.

Cases	Controls
**Inclusion criteria**	**Inclusion criteria**
1. Positive history and/or doctor-diagnosed moderate/severe AR	1. No personal and immediate family history of allergic diseases
2. Positive skin prick test (SPT) to HDM allergen	2. Negative SPT to HDM allergen
3. 18 years and older	3. 18 years and older
Note: Severity of AR is categorized based on the Allergic Rhinitis Impact on Asthma (ARIA) classification^9^	
**Exclusion criteria (cases and controls)**	
1. Subjects with autoimmune disease	
2. Subjects with immunocompromised or immunosuppressed condition including diabetes mellitus, malignant diseases and/or AIDS or HIV-positive	
3. Patients on long-term oral steroids or cytotoxic drugs	
4. Patients using anti-allergy agents including steroids, anti-histamine, and leukotriene receptor antagonists in the recent two months	
5. Pregnant	

Sample size calculation was conducted according to the difference of means between two independent groups using the software G*Power (version 3.1.9.3) based on past qPCR studies of nasal mucosa tissue samples^4,11,27,41,42^. A two-tailed hypothesis, α-error probability of 0.05, power (1-β error probability) of 0.80, effect size of 0.75, allocation ratio (N2/N1) of 1 and dropout rate of 5% were adopted. This yielded a total sample size of 60 participants divided equally between non-allergic controls and AR patients group (n = 30 per group). The recruitment of cases and controls of this study was conducted from March 2019 to July 2019.

### Data and samples collection

All the study participants were briefed about the study background and procedures by the study investigators. The patients were assessed with AR nasal and non-nasal symptoms severity scores. The nasal symptoms assessed were sneezing, runny or itchy nose, congestion (stuffiness) and postnasal drip. Non-nasal symptoms assessed were eye, throat or ear symptoms, chronic cough, headache and mental functions. We also assessed the global assessment of nasal and non-nasal symptoms severity and the QOL assessment of rhinitis severity. The 7-point visual analogue scale (VAS) was used in these assessments according to Spector *et al.*^43^ (Supplementary Table S3). All clinico-demographic data from each participant was obtained through a *Pro Forma* questionnaire (Supplementary Table S3). The data obtained from the *Pro Forma* consisting of demographic, clinical or study-related data (*i.e.* assessment from the patients), environmental factors, comorbidities and clinical findings.

### Skin prick test (SPT)

The study participants were screened using SPT to examine their sensitization towards HDMs (*D. farinae*, *D. pteronyssinus*, or *B. tropicalis*) allergens. This was to fulfil the study criteria before their samples were collected. The forearm of the participant was pricked using intradermal sterile lancet in five separate pricks. One drop of each allergen and control (histamine as positive control and saline as negative control) was placed at the pricked area. The pricked area was observed for about 15–30 min to detect the sensitization towards the allergens. The patients with wheal size of 4 mm and more was considered positive for sensitization and they were recruited to be in the cases group. The non-allergic control that showed no sensitization towards allergen was recruited to be in the controls group.

### Nasal epithelial cells collection

Nasal epithelial cells were collected by using cytology brush (Citotest Labware Co. Ltd, Haimen City, China). This nasal brushing was conducted by authorized physician in ORL-HNS clinic. The participants were instructed to clean their nostril beforehand. The brush was wet and sterile with isotonic saline solution (NaCl 0.9%). The brush was fully inserted into the nostrils and rubbed a few times rapidly against the medial and superior side of the inferior nasal meatus, using rotatory and linear movements. The brush was taken out and placed immediately into a 1.5 ml microcentrifuge tube containing 350 µl extraction buffer RLT (Qiagen, Hilden, Germany), supplemented with 1% (v/v) β-mercaptoethanol and was swirled to dislodge the cells (Supplementary Video S1).

The remaining liquid from the brush was removed by additional centrifugation of the microcentrifuge tube containing the brush. The remaining liquid was transferred into the microcentrifuge tube containing cell lysate. The tube containing the cell lysate was kept in − 80 °C until further use for RNA extraction. Cytology brush used for nasal epithelial cells sample collection and the picture of subject during nasal brushing procedures are shown in Fig. 2A,B, respectively.

### RNA extraction and reverse transcription-PCR (RT-PCR)

Frozen cell lysate was thawed before a total of 350 µl of 70% ethanol was added into the cell lysate and was mixed well by pipetting. RNA extraction was conducted using the RNeasy Mini Kit (Qiagen, Hilden, Germany) according to manufacturer’s protocols. The eluted RNA was then quantitated using BioTek Epoch microplate spectrophotometer (Epoch, BioTek, USA) and ratio of the absorbance at 260 nm and 280 nm (A260/280) as well as 260 nm and 230 nm (A260/230) were used to monitor the quality of extracted RNA. The ratio of A260/280 and A260/230 of the extracted RNA were at least 2.0 and 2.0–2.2, respectively. The extracted RNA was aliquoted and stored at − 80 °C until further use.

RT-PCR was performed using iScript Reverse Transcription (RT) Supermix for RT-qPCR (Bio-Rad, Philadelphia, PA, USA) and GeneAmp PCR System 9700 (Applied Biosystems, Waltham, USA) for incubation. Variable amount of RNA extracted from the nasal epithelial cells sample was used as the template for complementary DNA (cDNA) synthesis. Subsequently, the RNA was mixed with 4 μL of iScript RT Supermix provided by the kit. The mixture was then added with nuclease-free water (provided by the kit) to a final volume of 20 μL as the complete reaction mix. The complete reaction mix was then incubated in a thermal cycler using the following thermal profile: (1) Priming at 25 °C for 5 min; (2) Reverse transcription step at 46 °C for 20 min; (3) RT inactivation step at 95 °C for 1 min.

### Quantitative PCR (qPCR)

qPCR was performed using iTaq Universal SYBR Green Supermix (Bio-Rad, Philadelphia, PA, USA) and primers (Integrated DNA Technologies, Singapore) designed using NCBI Primer-BLAST (https://www.ncbi.nlm.nih.gov/tools/primer-blast/) as listed in Table 6. The following criteria were used in designing the primers:

1. Each forward or reverse primer to be 18–25 nucleotides in length;
2. The primer melting temperature (Tm) to be within 55–65 °C;
3. The GC-content (in percentage) to be less than 60% to avoid amplification of other GC-rich regions due to intrinsic lack of specificity of GC-rich regions;
4. For each gene, at least one of the two primers (forward or reverse) was designed to span an exon-exon junction to exclude the possibility of genomic DNA amplification;
5. The BLAST results of each primer pair was examined to ensure the absence of amplification of genes other than the gene of interest.

**Table 6:** List of primers used for SYBR Green qPCR.

Target gene	Accession number	Forward primer (5′-3′)	Reverse primer (5′-3′)	Amplicon size (bp)	Primer spans exon junction
*OCLN*	NM_002538.3	CGAGGAGTGGGTTAAAAATGTGTCT	GCTTGTCATTCACTTTGCCATT	122	Yes-Forward
*CLDN3*	NM_001306.4	CCACGCGAGAAGAAGTACACG	AGACGTAGTCCTTGCGGTCGTA	106	No^†^
*CLDN7*	NM_001307.6	TTTTCATCGTGGCAGGTCTTG	CCCTGCCCAGCCAATAAAGA	140	Yes-Forward
*TSLP*	NM_033035.5	GAAACTCAGATAAATGCTACTCAGG	TCAGTAAAGGTCGATTGAAGC	127	Yes-Forward
*DSG3*	NM_001944.3	AGTGCCTCAAACTCACTGGT	ACGGACTTCCCCAGTGTTTC	150	Yes-Forward
*GAPDH*	NM_002046.7	TCGGAGTCAACGGATTTGGT	TTCCCGTTCTCAGCCTTGAC	181	Yes-Forward

^†^*CLDN3* has only one exon, thus the primer was designed within the exon.

The MX3005P qPCR thermal cycler was used in the qPCR reaction (Agilent Technologies, Santa Clara, CA). The mixture for one qPCR reaction was prepared with 10 μL of iTaq Universal SYBR Green Supermix (2x) at final concentration of 1x, 2 μL (400 nM) each for forward and reverse primer, variable amount of cDNA template at final concentration of 50 ng and nuclease-free water was added into the mixture for a final volume of 20 μL. qPCR reaction was then performed according to the following thermal profile: (1) Polymerase activation step at 95 °C for 25 s; (2) Denaturation step at 95 °C for 5 s; (3) Annealing/extension step at 60 °C for 20 s. All three steps were repeated for 40 cycles. The primers were reconstituted to yield a 10 × concentration by resuspending in 950 µL of nuclease-free water followed by centrifugation at 2,500 rpm for 10 s before use. The relative transcripts quantity of every target gene in every sample was determined using the 2^−ΔΔCt^ formula whereby ΔΔCt = [(Ct sample − Ct control) − ΔCt1]..

### Statistical analysis

Data were analyzed using Student’s t-test (for normally distributed data) and Mann–Whitney U test (for not normally distributed data) to determine the difference of gene expression between AR and non-allergic control groups. The distribution of clinico-demographical and environmental parameters in AR patients or non-allergic controls were compared in terms of each gene’s expression (median cut-off) using the χ2-test or Fisher’s exact test as appropriate (GraphPad Prism v6.07; GraphPad Software Inc., CA, USA). All *p*-values were two-tailed and values < 0.05 were considered statistically significant.

## Supplementary information

Supplementary Video.

Supplementary Information.
