# Supplementary material for: Reduced occludin and claudin-7 expression is associated with urban locations and exposure to second-hand smoke in allergic rhinitis patients
Source: Sci Rep. 2021 Jan 13;11:1245. doi: 10.1038/s41598-020-79208-y (PMC7806883; doi:10.1038/s41598-020-79208-y)
Supplement: Supplementary file 2 — Supplementary Information. [file 41598_2020_79208_MOESM2_ESM.docx]

**Reduced occludin and claudin-7 expression is associated with urban locations and exposure to second-hand smoke in allergic rhinitis patients**

Siti Muhamad Nur Husna, Che Othman Siti Sarah, Hern-Tze Tina Tan, Norasnieda Md. Shukri, Noor Suryani Mohd Ashari, Kah Keng Wong

**Supplementary Table S1**: Nasal and non-nasal symptoms severity score in AR patients (n=30)

| **Nasal and non-nasal symptom severity scores (1-7)** | **AR patients** |
| --- | --- |
| **Nasal symptom** | **Median (IQR)** |
| Sneezing  Runny nose  Congestion (stuffiness)  Itchy nose  Postnasal drip | 5.0 (4.0-6.0)  5.0 (3.0-6.0)  5.0 (4.0-6.0)  5.0 (3.5-6.0)  4.5 (3.0-6.0) |
| **Non-nasal symptom** | **Median (IQR)** |
| Eye symptom  Throat symptom  Chronic cough  Ear symptom  Headache  Mental function | 4.0 (2.0-5.0)  3.0 (1.0-5.0)  2.0 (1.0-4.0)  4.0 (2.0-5.0)  4.0 (2.5-5.5)  4.0 (2.0-5.0) |

Abbreviation: IQR, interquartile range

**Supplementary Table S2**: The effects of nasal and non-nasal symptoms severity and rhinitis severity on QOL scores in AR patients (n=30)

| **Parameters** | **Quality of life (QOL) scores (1-7); Median (IQR)** |
| --- | --- |
| Effect of nasal and non-nasal symptom severity in the QOL scores of AR patients | 3.0 (2.0-4.5) |
|  | **Median (IQR)** |
| Effect of rhinitis severity in the QOL scores of AR patients | 3.0 (2.0-5.0) |

Abbreviation: IQR, interquartile range

**Supplementary Table S3:** *Pro Forma* questionnaire

| ID: |  |  |  |  |  |  |
| --- | --- | --- | --- | --- | --- | --- |

| Date: | d | d | **/** | m | m | **/** | y | y |
| --- | --- | --- | --- | --- | --- | --- | --- | --- |

**Important: Recruit has to sign his/ her consent form before answering this questionnaire**

| PART 1: PARTICIPANT DEMOGRAPHICS |
| --- |

| Registration no.: |  | Smoking : | Yes/ No |
| --- | --- | --- | --- |
| Sex: | ⬜ Male  ⬜ Female | Occupation: |  |
| Age: |  | Phone.: | M:  H: |
| Race :  Weight (kg):  Height (m):  BMI: | Malay/ Chinese/ Indian/ Others (specify: ) |  |  |
|  |  |  |  |
|  |  |  |  |
|  |  |  |  |
| Address : |  | | |

| **PART 2: CLINICAL DATA**  To be recruited as a healthy **control**, participant must answer questions 1-8 administered by a researcher/doctor, all with negative answers (i.e. ‘No’) |
| --- |

1. Having two or more of the following symptoms for more than one hour on most days:

⬜ watery anterior rhinorrhea ⬜ nasal obstruction

⬜ nasal pruritus ⬜ sneezing, especially paroxysmal

⬜ conjunctivitis ⬜ No

1. Personal history of chronic nasal/ sinus disease or frequent cold:

⬜ Yes ⬜ No

1. Personal history of allergy (e.g. allergic rhinitis/ hay fever, asthma, eczema, food allergy, urticaria):

⬜ Yes, specify: ⬜ No

1. Immediate family history of allergy:

⬜ Yes, please specify: ⬜ No

|  | Mother | Father | Bro1 | Bro2 | Sister 1 | Sister 2 | Other Bro | Other sister |
| --- | --- | --- | --- | --- | --- | --- | --- | --- |
| Asthma |  |  |  |  |  |  |  |  |
| Dermatitis |  |  |  |  |  |  |  |  |
| Eczema |  |  |  |  |  |  |  |  |
| Hay Fever |  |  |  |  |  |  |  |  |
| Seasonal allergies |  |  |  |  |  |  |  |  |
| Year round allergies |  |  |  |  |  |  |  |  |
| Sinus problem |  |  |  |  |  |  |  |  |
| Lupus |  |  |  |  |  |  |  |  |
| Arthritis |  |  |  |  |  |  |  |  |

1. Have autoimmune disorder:

⬜ Yes, specify: ⬜ No

1. Are you immunocompromised or immunosuppressed (e.g. diabetes mellitus, malignant diseases, patients on long term oral steroids or cytotoxic drugs, AIDS, HIV positive):

⬜ Yes, specify: ⬜ No

1. Having fever:

⬜ Yes ⬜ No

1. a) Are you **currently** taking any medication? b) Have you **previously** taken any medication

⬜ Yes, specify: ⬜ Yes, specify:

⬜ antihistamine: ⬜ antihistamine:

⬜ nasal spray: ⬜ nasal spray:

⬜ others: ⬜ others:

⬜ No ⬜ No

1. Are you pregnant:

⬜ Yes ⬜ No

| Participant recruited as **control** to skip answering questions below of part 2 to part 6 (for patients only). |
| --- |

1. Duration of illness (AR/ allergies):
2. Pruritus (itching):

⬜ Yes ⬜ No

**Severity of allergic rhinitis symptoms:**

Sleep ⬜ Normal ⬜ Abnormal

Daily activities ⬜ Normal ⬜ Impaired

Problem caused at work/place ⬜ No ⬜ Yes

Troublesome symptoms ⬜ No ⬜ Yes

**Diagnosis**: ⬜ Mild allergic rhinitis

⬜ Moderate/severe allergic rhinitis

**Classification**: ⬜ Intermittent

⬜ Persistent

**PART 3: ASSESSMENT OF NASAL SYMPTOM SEVERITY**

Please rate the following nasal symptoms by circling a number on each line according to severity:

| 1 | 2 | 3 | 4 | 5 | 6 | 7 |
| --- | --- | --- | --- | --- | --- | --- |
| 1 | 2 | 3 | 4 | 5 | 6 | 7 |
| 1 | 2 | 3 | 4 | 5 | 6 | 7 |
| 1 | 2 | 3 | 4 | 5 | 6 | 7 |
| 1 | 2 | 3 | 4 | 5 | 6 | 7 |

Sneezing

Runny nose

Congestion

Itchy nose

Postnasal drip

Key to symptoms (according to Spector *et. al* ^43^):

| 1 | None – to an occasional limited episodes |
| --- | --- |
| 2 |  |
| 3 | Mild – steady symptoms but easily tolerable |
| 4 |  |
| 5 | Moderately bothersome – symptoms hard to tolerate, may interfere with activities of daily living and/ or sleep |
| 6 |  |
| 7 | Unbearable severe- symptoms are so bad, person can’t function all the time |
|  |  |

**PART 4: ASSESSMENT OF NON-NASAL SYMPTOM SEVERITY**

Please rate the following non-nasal symptoms by circling a number on each line according to severity:

| 1 | 2 | 3 | 4 | 5 | 6 | 7 |
| --- | --- | --- | --- | --- | --- | --- |
| 1 | 2 | 3 | 4 | 5 | 6 | 7 |
| 1 | 2 | 3 | 4 | 5 | 6 | 7 |
| 1 | 2 | 3 | 4 | 5 | 6 | 7 |
| 1 | 2 | 3 | 4 | 5 | 6 | 7 |
| 1 | 2 | 3 | 4 | 5 | 6 | 7 |

Eye symptoms

Throat symptoms

Chronic cough

Ear symptoms

Headache

Mental function

Key to symptoms (according to Spector *et. al* ^43^):

| 1 | None – to an occasional limited episodes |
| --- | --- |
| 2 |  |
| 3 | Mild – steady symptoms but easily tolerable |
| 4 |  |
| 5 | Moderately bothersome – symptoms hard to tolerate, may interfere with activities of daily living and/ or sleep |
| 6 |  |
| 7 | Unbearable severe- symptoms are so bad, person can’t function all the time |
|  |  |

**PART 5: GLOBAL ASSESSMENT OF NASAL AND NON-NASAL SYMPTOM SEVERITY**

Please rate the quality of person’s life by circling a number on the appropriate place on the line:

How does the person generally feel in terms of his/her nasal and non-nasal symptoms?

High numbers mean he/she is feeling the best.

| 1 | 2 | 3 | 4 | 5 | 6 | 7 |
| --- | --- | --- | --- | --- | --- | --- |

Severely affected Excellent

**PART 6: QUALITY OF LIFE ASSESSMENT OF RHINITIS SEVERITY**

Please rate the quality of person’s life by circling a number on the appropriate place on the line:

| 1 | 2 | 3 | 4 | 5 | 6 | 7 |
| --- | --- | --- | --- | --- | --- | --- |

Severely affected Excellent

Key (according to Spector *et al.* ^43^):

| 1 | Quality of life is **terribly affected** in terms of sleep disturbance at night and/ or impairment of work performance and/ or impairment of social and/ or recreational activities. |
| --- | --- |
| 2 | Quality of life is **affected almost all the time** in terms of sleep disturbance at night and/ or impairment of work performance and/ or impairment of social and/ or recreational activities. |
| 3 | Quality of life is **affected** **often** in terms of sleep disturbance at night and/ or impairment of work performance and/ or impairment of social and/ or recreational activities. |
| 4 | Quality of life is **affected occasionally** **but it is tolerable** in terms of sleep disturbance at night and/ or impairment of work performance and/ or impairment of social and/ or recreational activities. |
| 5 | Quality of life is **hardly affected** in terms of sleep disturbance at night and/ or impairment of work performance and/ or impairment of social and/ or recreational activities. |
| 6 | Quality of life is **so mildly affected it is hardly noticed** in terms of sleep disturbance at night and/ or impairment of work performance and/ or impairment of social and/ or recreational activities. |
| 7 | **Excellent quality of life** in terms of sleep disturbance at night and/ or impairment of work performance and/ or impairment of social and/ or recreational activities. |

| **PART 7: ENVIRONMENTAL FACTORS** |
| --- |

1. Does anyone in your house smoke cigarettes?

⬜ Yes - go to Question 2 & 3 ⬜ No - go to Question 4

1. How many people living in the house smoke cigarettes? ⬜ people
2. About how many cigarettes does he/ she smoke each day? ⬜ number of cigarettes
3. Which of the following places have you been exposed to secondhand smoke?

⬜ at home ⬜ in the vehicle ⬜ other - please specify………..

⬜ work place ⬜ public places ⬜ No

1. How often have you been exposed to second-hand smoke?

⬜ daily ⬜ weekly ⬜ monthly ⬜ never

1. Home location

⬜ Urban area ⬜ Rural area

1. Are you living in an industrial area and exposed to smoke?

⬜ Yes ⬜ No

1. In the past 12 months, have you had pets at home?

⬜ Yes - please specify below ⬜ No

Inside outside both

Cat ⬜ ⬜ ⬜

Dog ⬜ ⬜ ⬜

Bird ⬜ ⬜ ⬜

Other ⬜ ⬜ ⬜

1. Do you have regular contact with farm animals (*e.g.* cattle, goats, sheep or poultry)?

⬜ Yes ⬜ No

1. How frequent do you change bed sheets and pillowcases?

⬜ weekly ⬜ monthly ⬜ 2-monthly ⬜ other- please specify ……….

1. How frequent do you do housekeeping?

⬜ daily ⬜ every alternate day ⬜ weekly ⬜ other-please specify ……….

| **PART 8: OTHER CO-MORBIDITIES** (Researcher to obtain data from patient’s folder) |
| --- |

| ⬜ Conjunctivitis | ⬜ Otitis media | ⬜ Sinusitis |
| --- | --- | --- |
| ⬜ Pharyngitis | ⬜ Asthma | ⬜ Speech impairment |
| ⬜ Lymphoid hypertrophy/ obstructive sleep apnea | | |

**PART 9: SKIN PRICK TEST**

| **Allergens** | **Wheal size (mm)** |
| --- | --- |
| Positive Control |  |
| Negative Control |  |
| *Blomia tropicalis* |  |
| *Dermatophagoides farinae* |  |
| *Dermatophagoides pteronysinnus* |  |

**PART 10: CLINICAL FINDING**

Anterior rhinoscopy

1. Inferior turbinate:

Size of hypertrophy: ⬜ 1 ⬜ 2 ⬜ 3

Colour: ⬜ Pale ⬜ Normal ⬜ Inflammed

2. Presence of discharge: ⬜ Yes ⬜ No

-END OF SURVEY-
